# The Book World of Medicine and Science

**Published:** 1895-08-10

**Authors:** 


### The Book World of Medicine and Science.

Rainmaking and Sunshine, by John Collinson. London: Swan Sonnenschein and Co., 1894. Price 3s. 6d.

This little book, published in the year of grace 1894, has recently come into our hands, and makes us think that wit must be as various as weather in the British Isles if within their confines readers can be found for such effusions.

It would seem that, according to the author, there is in nature a hitherto unrecognized force called odyle, and that there dwell upon this earth people who are so susceptible to this force and have such power over it as to merit the name of "sensitives." These people are evidently uncanny folk, and their power is immense; for example, "od or odyle when properly used by a sensitive under proper conditions, can act so that the sun can be so influenced that it will shine brilliantly day after day as may be arranged. What an important force it is, and must be!" Truly it must!

The author after experiments and observations finds that he is a "sensitive," and modestly describes his powers over the elements. "They are simply marvellous. Storms, floods, drought, &c., can be induced on the one hand, and the prevalence of sunshine and warmth, in opposition to coldness and gloom, on the other. His action in this direction, judging from experience, could bring any district, and, indeed, the country generally, such favourable weather as would recall the glories of the Golden Age."

It is perhaps fortunate for the author that he lives in an age of little faith and many policemen, for we fear that, in times not so long gone by, anyone who ostentatiously took on himself such responsibility in regard to the vagaries of the English climate would have received small mercy from those who believed his assertions. It seems, however, that the "sensitive" is not always to be blamed when things go wrong, for to ensure success his surroundings should be quiet and peaceful. "Noise and turmoil, knocking, and exploding of fog signals, the slamming of railway carriage doors, the letting off of firearms or fireworks, the beating of carpets and drums, the playing of brass bands near to him, and within hearing—in fact, any violence or concussion which would put the air in active motion within the odylic circumference of the sensitive would cause rain and wind to be sure to follow." The wonder is that we ever get fine weather at all in noisy towns, for we are told that "the noise of a Salvation Army drum has often spoilt most charming weather."

Of course, the difficulty is that if the "sensitive" is kept too quiet and retired, the weather becomes too dry. This, however, is soon put right; a run up to London, and the exposure of the "sensitive" to the noise and turmoil thereof will soon bring rain again. "The writer can prove that he went to London, where there was plenty of noise, and motion, and tumult, on purpose, twice—by arrangement—in 1893, and on each occasion broke the drought, May 15th, and June 19th, when there; and there need be no serious drought at any time if the officials of the Government had the good judgment to enlist the services of this extraordinary force."

We are sorry for the writer of such a rigmarole, but what are we to say about his publisher?

Clinical Lectures on the Prevention of Consumption. By William Murrell, M.D., F.R.C.P. (London: Baillière, Tindal, and Cox. 1895. Pp. 103)

Dr. Murrell's pleasant and easy style of writing is well known, and is particularly well exemplified in the manner in which he handles the various statistics bearing on the ætiology, treatment, and prophylaxis of consumption. He has brought together most convincing evidence of the contagiousness of phthisis, and the pernicious influence of various adverse hygienic conditions which predispose to its occurrence. The language is clear, concise, and to the point, and its eminently practical character will render this book most useful, and well worth reading by heads of families, schools, and institutions, as well as by practitioners of medicine, for no intelligent man or woman can afford to remain ignorant of the simple and elementary yet woefully neglected principles which should guide us in avoiding contagion by this ubiquitous disease.

